# The Factors Influencing the Decision to Pursue Orthopaedic Surgery Residency Vary by Race and Gender: A Survey-Based Study

**DOI:** 10.7759/cureus.86490

**Published:** 2025-06-21

**Authors:** Chrystina L James, Johnny Kasto, Mahdi Mazeh, Ryan Sanii, Gabriel Burdick, Bushra Fathima, Eric Jiang, Stephanie Muh

**Affiliations:** 1 Department of Orthopaedic Surgery, Henry Ford Health System, Detroit, USA; 2 Department of Orthopaedic Surgery, University of Southern California, Los Angeles, USA; 3 Department of Neurosurgery, Yale School of Medicine, New Haven, USA; 4 Department of Orthopaedic Surgery, Henry Ford Health System, West Bloomfield, USA

**Keywords:** gender, medical student, orthopaedic surgery, race, residency, resident

## Abstract

Background and objective

Despite ongoing efforts to increase diversity in the field, orthopaedic surgery continues to be the least diverse specialty in all of medicine. This study aimed to assess experiences in medical school and their impact on the decision to pursue orthopaedic surgery residency, and specifically, if these experiences varied by race, including underrepresented minority (URM), or gender. We hypothesized that male and Caucasian residents would report earlier exposure to orthopaedics and mentorship, contributing to earlier decisions to pursue the field.

Methods

A voluntary survey assessing factors influencing the decision to pursue orthopaedic surgery was sent to 2,122 orthopaedic surgery residents. We compared differences in response between male and female genders as well as three different racial groups (URM, Asian, and Caucasian). Differences in ordinal variables between independent groups were compared using independent t-tests for normally distributed data and Mann-Whitney U tests for non-normally distributed data. Differences in categorical data were analyzed using the χ2 test or Fisher's exact test as appropriate.

Results

A total of 337 residents completed the survey, yielding a response rate of 15.9%. Males were more likely than females to agree that a role model or mentor of the same sex and race positively influenced their decision [median interquartile range: (IQR): 4 (2-5) vs. 2 (2-4); Mann-Whitney U=8807, p<0.001, r=0.32] and were more likely to disagree they experienced gender-based discrimination [1 (1-2) vs. 3 (2-4); U=7834, p<0.001, r=0.38). Interest in orthopaedics before medical school was higher in males (n=145, 64.7%) than females (n=56, 49.6%), while 15.0% (n=17) of females became interested during elective rotations compared to 7.1% (n=16) of males [χ²(3, N=337)=9.10, p=0.028, V=0.16]. More Caucasian residents (n=166, 64.3%) became interested in orthopaedic surgery before medical school compared to URM (n=22, 57.9%) and Asian (n=13, 31.7%) residents, while 7.7% (n=20) of Caucasians became interested during elective rotations compared to 12.2% (n=5) of Asians and 21.0% (n=8) of URM residents [χ²(6, N=337)=25.11, p<0.001, V=0.22].

Conclusions

There are several significant differences in the experiences of female and URM medical students who chose to pursue orthopaedic surgery relative to male and Caucasian students. The low response rate may reflect self-selection bias and should be taken into account when interpreting these findings.

## Introduction

Racial and gender diversity in medical school has drastically increased in recent years, with female and non-Caucasian students making up the majority of medical students since 2019 and 2020, respectively [1]. However, orthopaedic surgery residents remain predominantly male and Caucasian. In 2022, 7-8% of orthopaedic surgeons and 18.3% of orthopaedic surgery residents identified as female; 72.8% of residents identified as white, 13.9% as Asian, and 5.7% as black or African American [2-4]. A recent study found that from 2006 to 2015, there was a significant decrease in racial diversity among orthopaedic surgery residents [5]. From 2002 to 2016, there was a significant increase in the number of orthopaedic residencies, albeit without a single underrepresented minority (URM) resident [6]. It remains unclear whether this trend has reversed in more recent years, making contemporary assessments such as ours essential for identifying ongoing disparities.

Many reasons have been proposed for this continued lack of diversity, including a lack of mentorship and early exposure to the field [7-9]. Several studies have demonstrated the importance of mentorship for medical students in deciding what specialty to pursue. Exposure to the field of orthopaedic surgery has also been shown to be important in increasing diversity within orthopaedics and can be facilitated via a medical school musculoskeletal (MSK) education curriculum; however, less than half of all medical schools have required MSK education [10,11]. In addition to formal curricula in MSK medicine, programs like Nth Dimension and the Perry Initiative have been developed to provide mentorship and early exposure to the specialty of orthopaedics to underrepresented students. These programs have been shown to increase interest in orthopaedics among participants and have led to these students applying and matching into orthopaedic surgery at higher rates [12,13].

There is a common perception among medical students that orthopaedic surgery is not “female-friendly” and is not conducive to having or raising a family. Many female trainees cite concerns regarding pregnancy, maternity leave, breastfeeding policies, and the growing evidence of increased risk of peripartum complications among pregnant surgeons [14-17]. Female students are more likely than males to view orthopaedics as male-dominated and requiring greater physical strength, while both male and female students feel that orthopaedics is associated with long work-hours and lacks an achievable work-life balance or family-friendly practice [7,18]. Female and URM medical students also perceive orthopaedics to be less diverse and inclusive than male and racial majority students; they feel that they will have to work harder to be recognized, and face frequent microaggressions in the workplace [19-23].

The purpose of this study was to examine whether key medical school experiences - such as mentorship, exposure to orthopaedic surgery, and experiences with discrimination - influence the decision to pursue orthopaedic surgery residency, and whether these experiences differ by race and gender. The specific aims of this study were as follows: (1) to evaluate the timing and influence of mentorship, clinical exposure, and research involvement on the decision to pursue orthopaedics; (2) to determine whether these experiences vary across racial and gender groups; and (3) to assess the prevalence of perceived discrimination during orthopaedic surgery rotations in medical schools. We hypothesized that males and Caucasians would have earlier exposure to and interest in orthopaedics and, therefore, earlier mentorship and research exposure.

## Materials and methods

A voluntary survey was designed to assess factors influencing the decision to pursue orthopaedic surgery and was distributed to all orthopaedic surgery residents listed in the American Orthopaedic Association (AOA) Council of Orthopaedic Residency Directors (CORD) contact list. The survey was reviewed by a multidisciplinary team of orthopaedic educators and residents to ensure face validity and content relevance. The survey aimed to gather demographic information, training background, and perceptions regarding mentorship, discrimination, and exposure to orthopaedic surgery during medical school. The survey was electronically distributed to all 2,122 orthopaedic surgery residents in the AOA CORD database, which includes residents from a broad range of residency programs across the United States. A follow-up reminder email was sent two months later to residents who had not yet completed the survey. Participation was voluntary, and respondents who only completed the demographic section were excluded from the analysis. While the AOA CORD database includes the majority of orthopaedic surgery residency programs in the US, it may not encompass all programs or residents. Therefore, the surveyed population may not be fully representative of the national orthopaedic resident cohort.

The survey included questions regarding self-reported demographic and residency training information, such as gender, race, sex, postgraduate year (PGY) level, and the geographical location of their residency program. The American Association of Medical Colleges (AAMC) definition of Underrepresented in Medicine (URM) was used to classify race and ethnicity. Specifically, residents who self-identified as black or African American, native Hawaiian or other Pacific Islander, or “Other” were categorized as URM for the purposes of this study.

Survey questions were designed to evaluate key factors influencing career selection, including the role of mentorship, early exposure to the field, perceived discrimination, and the perceived inclusivity of the orthopaedic surgery specialty. Residents were asked about their experiences with mentorship, their perceptions of gender and racial diversity within the field, and their experiences with discrimination and microaggressions. In addition, respondents were asked when they first became interested in orthopaedic surgery, when they identified a mentor in the field, and when they became involved in orthopaedic-related research. The survey instrument contained a mix of ordinal and nominal questions. Responses to questions regarding the impact of mentorship, discrimination, and faculty perceptions were rated on a five-point Likert scale (1=Strongly Disagree, 5=Strongly Agree). Responses to questions regarding the timing of exposure to orthopaedics and research involvement were categorical and analyzed accordingly.

Descriptive statistics were calculated to summarize the collected data, including mean, median, standard deviation (SD), and interquartile range (IQR). Ordinal variables were assessed for normality using the Shapiro-Wilk test. Normally distributed continuous variables were reported as mean ± SD, while non-normally distributed variables were represented as median (IQR). To compare differences between male and female respondents as well as between racial groups (URM, Asian, and Caucasian), all ordinal variables were first assessed for normality using the Shapiro-Wilk test. As most ordinal variables did not follow a normal distribution, comparisons were made using the Mann-Whitney U test. Categorical variables were analyzed using the chi-square (χ²) test or Fisher’s exact test as appropriate. Test statistics, including degrees of freedom (df) and effect sizes (Cramer’s V or phi coefficient), were reported. Statistical significance was set at a p-value threshold of <0.05.

All statistical analyses were performed using JMP® software (Version 17.2.0, SAS Institute Inc., Cary, NC, 1989-2022). The study was conducted per ethical research guidelines, and data collection was performed anonymously to ensure confidentiality. Since the study involved voluntary survey responses without patient data, the institutional review board (IRB) approval was not required.

## Results

This survey was distributed to 2,122 orthopaedic surgery residents via the AOA CORD database; 337 residents completed the survey in its entirety, yielding a response rate of 15.9%. Respondents self-identified as white (76.6%, n=258), Asian (12.2%, n=41), and URM (11.2%, n=38). Of those considered URM for this study, 17 identified as black or African American, two as native Hawaiian or other Pacific Islander, and 19 as other. Of note, 66.5% (n=224) of the respondents were male and 33.5% (n=113) female. The PGY standing of respondents was as follows: 14.8% PGY-1 (n=50), 13.3% PGY-2 (n=45), 16.6% PGY-3 (n=56), 25.2% PGY-4 (n=85), and 29.4% PGY-5 (n=99); 0.6% (n=2) did not respond to this question. Most were at residency programs located in the Northeast (36.5%, n=123) and Midwest (28.8%, n=97), with a smaller number in the Southeast (16.9%, n=57), West (9.8%, n=33), and Southwest (8.0%, n=27). 

As shown in Table 1, male and female residents had similar levels of agreement that a role model or mentor in orthopaedic surgery positively influenced their decision to go into orthopaedic surgery; however, males were significantly more likely than females to agree that a role model or mentor of the same sex and race positively influenced their decision to enter the field: median (IQR): 4 (2-5) for males and 2 (2-4) for females (Mann-Whitney U=8807, p<0.001, r=0.32). Males were also significantly more likely to disagree that they had experienced gender-based discrimination during an orthopaedic surgery rotation in medical school: 1 (1-2) for males vs. 3 (2-4) for females (U=7834, p<0.001, r=0.38). There were no significant differences based on gender in response to questions regarding perceived happiness of orthopaedic faculty, involvement in research, race-based discrimination, or feeling respected by residents and faculty during orthopaedic surgery rotations.

**Table 1:** Survey responses by gender ^*^Statistically significant Data are depicted as response (1–5), median (IQR) IQR: interquartile range

Question	Female (n=113)	Male (n=224)	P-value	
1. A role model of a mentor in orthopaedic surgery positively influenced my decision to go into the field	5 (4–5)	4 (4–5)		0.536
2. A role model or mentor in orthopaedic surgery of the same sex and race positively influenced my decision to go into the field	2 (2–4)	4 (2–5)		<0.001^*^
3. The perceived happiness of the orthopaedic faculty positively influenced my decision to go into the field	4 (4–5)	4 (4–5)		0.6
4. My involvement in orthopaedic surgery research positively influenced my decision to go into the medical field	3 (2–4)	3 (2–4)		0.62
5. I experienced discrimination based on my gender during an orthopaedic surgery rotation in medical school	3 (2–4)	1 (1–2)		<0.001^*^
6. I experienced discrimination based on my race during an orthopaedic surgery rotation in medical school	2 (1–2)	1 (1–2)		0.246
7. I felt respected by residents and faculty during orthopaedic surgery rotations in medical school	4 (2–4)	3 (1–4)		0.079

Table 2 summarizes the responses by race. Both Asian and Caucasian residents (n=41, 12.2% and n=258, 76.6%, respectively) agreed that a mentor of the same sex and same race influenced their decision [median (IQR): 4 (2-5)], while URM residents (n=38, 11.2%) disagreed [2.5 (1-4); χ²(2, n=337)=9.89, p=0.007, Cramer’s V=0.17]. For race-based discrimination, URM and Asian residents had higher agreement than Caucasians [2 (1-3) vs. 2 (1-3) vs. 1 (1-2); χ²(2, n=337)=13.38, p=0.001, V=0.20]. No racial differences were found in other areas.

**Table 2:** Survey responses by race *Statistically significant Data are depicted as response (1–5), median (IQR) IQR: interquartile range; URM: underrepresented minority

Question	URM (n=38)	Asian (n=41)	Caucasian (n=258)	P-value
1. A role model of a mentor in orthopaedic surgery positively influenced my decision to go into the field	4 (3–5)	5 (4–5)	4 (4–5)	0.166
2. A role model or mentor in orthopaedic surgery of the same sex and race positively influenced my decision to go into the field	2.5 (1–4)	4 (2–5)	4 (2–5)	0.007^*^
3. The perceived happiness of the orthopaedic faculty positively influenced my decision to go into the field	4 (3.75–5)	4 (4–5)	4 (4–5)	0.399
4. My involvement in orthopaedic surgery research positively influenced my decision to go into the medical field	3 (2–4)	3 (2–4)	3 (2–4)	0.925
5. I experienced discrimination based on my gender during an orthopaedic surgery rotation in medical school	2 (1–2.5)	2 (1–2)	2 (1–3)	0.427
6. I experienced discrimination based on my race during an orthopaedic surgery rotation in medical school	2 (1–3)	2 (1–3)	1 (1–2)	0.001^*^
7. I felt respected by residents and faculty during orthopaedic surgery rotations in medical school	4 (1–4)	4 (1.5–4)	3 (1–4)	0.69

There were no significant differences in terms of gender (Table 3) or race (Table 4) as to which stage/time period in medical school the respondents got involved in research or identified a mentor or role model in orthopaedic surgery. However, there were significant differences as to when respondents became interested in orthopaedic surgery, with more males developing an earlier interest than females. Of note, 64.7% (n=145) of males were interested in orthopaedic surgery before starting medical school relative to 49.6% (n=56) of females. In contrast, 15.0% (n=17) of females became interested in orthopaedics during their elective rotations compared to only 7.1% (n=16) of males [χ²(3, n=337)=9.10, p=0.028, V=0.16]. A higher proportion of Caucasian residents (64.3%, n=166) became interested in orthopaedic surgery before medical school than URM (57.9%, n=22) or Asian (31.7%, n=13) residents, while only 7.7% (n=20) of Caucasians became interested in orthopaedics during elective rotations compared to 12.2% (n=5) of Asians and 21.0% (n=8) of URM residents [χ²(6, N=337)=25.11, p<0.001, V=0.22].

**Table 3:** Responses to medical school-related questions by gender ^*^Statistically significant Data are depicted as the number of responses (%)

Question	Female (n=113)	Male (n=224)	P-value
When did you become interested in orthopaedic surgery?			0.028^*^
Before medical school	56 (49.6%)	145 (64.7%)	
Preclinical education	20 (17.7%)	35 (15.6%)	
Core clerkships	20 (17.7%)	28 (12.5%)	
Elective rotations	17 (15.0%)	16 (7.1%)	
When did you become involved in research in medical school?			0.516
1st year	61 (54.0%)	115 (51.4%)	
2nd year	22 (19.5%)	50 (22.3%)	
3rd year	20 (17.7%)	33 (14.7%)	
4th year	6 (5.3%)	9 (4.0%)	
I did not participate in research during medical school	4 (3.5%)	17 (7.6%)	
When did you identify a mentor or role model in orthopaedic surgery during medical school?			0.871
1st year	36 (31.9%)	78 (34.8%)	
2nd year	17 (15.0%)	38 (17.0%)	
3rd year	34 (30.1%)	63 (28.1%)	
4th year	12 (10.6%)	17 (7.6%)	
I did not have a mentor or role model in the field	14 (12.4%)	28 (12.5%)	

**Table 4:** Responses to medical school-related questions by race ^*^Statistically significant Data are depicted as the number of responses (%) URM: underrepresented minority

Question	URM (n=38)	Asian (n=41)	Caucasian (n=258)	P-value
1. When did you become interested in orthopaedic surgery?				0.001^*^
Before medical school	22 (57.9%)	13 (31.7%)	166 (64.3%)	
Preclinical education	6 (15.8%)	13 (31.7%)	36 (14.0%)	
Core clerkships	2 (5.3%)	10 (24.4%)	36 (14.0%)	
Elective rotations	8 (21.0%)	5 (12.2%)	20 (7.7%)	
2. When did you become involved in research in medical school?				0.201
1st year	19 (50.0%)	22 (53.7%)	135 (52.3%)	
2nd year	9 (23.7%)	5 (12.2%)	58 (22.5%)	
3rd year	3 (7.9%)	9 (22.0%)	41 (15.9%)	
4th year	2 (5.3%)	4 (9.7%)	9 (3.5%)	
I did not participate in research during medical school	5 (13.1%)	1 (2.4%)	15 (5.8%)	
3. When did you identify a mentor or role model in orthopaedic surgery during medical school?				0.648
1st year	12 (31.6%)	14 (34.2%)	88 (34.1%)	
2nd year	4 (10.5%)	7 (17.1%)	44 (17.1%)	
3rd year	10 (26.3%)	14 (34.2%)	73 (28.3%)	
4th year	5 (13.2%)	1 (2.4%)	23 (8.9%)	
I did not have a mentor or role model in the field	7 (18.4%)	5 (12.2%)	30 (11.6%)	

## Discussion

This study demonstrates the importance of mentorship in specialty selection for medical students, as most survey respondents agreed or strongly agreed that a mentor in orthopaedic surgery positively influenced their decision to pursue the field. In a previous survey of orthopaedic surgeons at academic programs, 84.2% felt that a mentor had been influential in their decision to apply to orthopaedic surgery residency [24]. More specifically, the present study found no difference based on race or gender as to when orthopaedic surgery mentors or role models were first identified. For male and Asian or Caucasian residents, these mentors were generally of the same race or gender. However, female and URM residents disagreed that their orthopaedic surgery mentors were race- or gender-concordant. A survey of medical students found that female students are more likely than males to identify having a role model of the same sex as being important in their decision to pursue orthopaedics [18,25]. Additionally, both female and URM medical students are more likely to apply to orthopaedic residency if they are at an institution with higher female and URM representation in orthopaedic surgery faculty and residents, again highlighting the importance of mentorship [26,27]. This highlights the importance of outreach programs and pipeline programs that expose students to these mentors and may explain some of the success these programs have had. 

Male medical students often have earlier exposure to orthopaedic surgery and thus decide to enter the field earlier, allowing more time for research, networking, and academic preparation. Hill et al. showed that males are more likely to choose orthopaedic surgery before beginning their clinical rotations, whereas females are more likely to choose it during rotations [25]. Our findings suggest that males and Caucasian respondents more frequently reported interest in orthopaedic surgery before beginning medical school; however, given the cross-sectional nature of this study, no conclusions about causality can be drawn. In contrast, female and URM respondents more commonly reported developing an interest during elective rotations. This pattern highlights the potential importance of clinical exposure in informing specialty choice for these groups. Delayed exposure, however, may limit the time available to identify mentors and strengthen residency applications in an increasingly competitive field. As discussed previously, mandatory medical school MSK education is one means of earlier exposure. A study by Bernstein et al. found that required education in MSK medicine significantly increased application rates to orthopaedic surgery residency by 75% in females and 35% in minorities [28].

In this study, male and Caucasian residents strongly disagreed that they experienced gender- or race-based discrimination during their medical school orthopaedic surgery rotations. Female, Asian, and URM residents, however, had higher levels of agreement that they had experienced this type of discrimination. This is consistent with previous research demonstrating that female and URM orthopaedic surgeons experience high levels of discrimination, harassment, and microaggressions at every level of practice. In one recent study, 96% of black orthopaedic surgeons reported experiencing workplace discrimination during training, with females reporting higher levels of discrimination than males [20,23]. In a survey of Ruth Jackson Orthopaedic Society members, 68% of female respondents reported experiencing sexual harassment during training [29]. An AAOS survey of members found that 81% of female respondents reported discrimination, bullying, and harassment in the workplace [22]. While most of the research into these experiences has focused on practicing orthopaedic surgeons, the results of the current study suggest that medical students are facing similar discrimination within orthopaedic surgery. This is an area that should be investigated further [30].

Limitations

The primary limitation of this study is the low response rate, resulting in a relatively small sample size; however, the response rate was similar to other previously published studies. Surveys were distributed to residents via the AOA CORD database, while not every orthopaedic surgery residency program is represented in the database, a majority are, and thus, a representative population was queried. As such, selection bias may influence the generalizability of our findings, and respondents may differ from the broader resident population in ways that affect survey responses. Females and URMs are over-represented in this study population relative to the percentage of female and URM orthopaedic residents; residents who chose to complete the survey may have had different experiences in medical school than those who did not complete it, resulting in response bias. This over-representation may amplify the prevalence of reported discrimination and perceptions of inclusivity or mentorship, and should be considered when interpreting group comparisons.

There may also be recall bias, as residents were asked to answer questions regarding their previous experiences as medical students. This is particularly relevant for questions involving timing-such as when respondents first became interested in orthopaedics, identified mentors, or began research, as recollections may be inaccurate or influenced by subsequent experiences. It is also important to consider the potential influence of social desirability bias, particularly among male respondents, which may have led to underreporting of discrimination experiences and could partially account for the observed gender differences. While this study categorized racial groups broadly, the aggregation of URM respondents may overlook important differences among subgroups (e.g., Black, Pacific Islander, 'Other'). Due to small subgroup sample sizes, further disaggregation was not feasible in this analysis. Future research with larger and more balanced samples is needed to explore these subgroup differences in greater detail.

## Conclusions

Our findings highlight several significant differences in the experiences of female and URM medical students who chose to pursue orthopaedic surgery, relative to male and Caucasian students. Increased and earlier exposure to the field through formal medical school MSK educational curricula, as well as early exposure programs, may encourage more students from diverse backgrounds to consider a career in orthopaedics. Increased availability of race- and gender-concordant mentors may help foster greater interest in orthopaedic surgery among underrepresented students; however, further prospective studies are required to determine this association's true impact on increasing diversity within the field. As such, we must continue to address the prevalence of discrimination, bias, and microaggressions that female and minority surgeons face regularly. It is important to recognize that not only are medical students observing these exclusionary interactions within our field, but they are also experiencing them firsthand. Identifying these differential experiences provides insights into areas to focus further research and efforts to increase diversity. Future studies may benefit from qualitative interviews to explore the nuanced personal experiences of underrepresented trainees, as well as longitudinal designs that track mentorship, exposure, and specialty choice over time to better understand causative relationships.
